# Effects of a TeamSTEPPS‐Based Patient Safety Workshop on Undergraduate Nursing Students' Teamwork Perceptions and Communication‐Related Knowledge

**DOI:** 10.1002/nop2.70825

**Published:** 2026-09-11

**Authors:** Yunrui Yin, Weirong Tang, Yuerong Li, Huilin Ye, Yingjia Zhang, Xiaorong Wu, Lulu Wan, Enqi Huang

**Affiliations:** ^1^ Department of Anesthesiology The First Affiliated Hospital of Chongqing Medical University Chongqing China; ^2^ Department of Gastroenterology The First Affiliated Hospital of Chongqing Medical University Chongqing China; ^3^ Department of Emergency The Second Affiliated Hospital of Chongqing Medical University Chongqing China; ^4^ Department of Otolaryngology The First Affiliated Hospital of Chongqing Medical University Chongqing China; ^5^ Department of Nursing The First Affiliated Hospital of Chongqing Medical University Chongqing China

**Keywords:** communication, nursing education, patient safety, TeamSTEPPS, teamwork, workshop

## Abstract

**Aim:**

To examine changes in undergraduate nursing students' teamwork perceptions and communication‐related knowledge following a 4‐h TeamSTEPPS‐based patient safety workshop.

**Design:**

A single‐group pretest‐posttest quasi‐experimental study.

**Methods:**

Third‐year nursing students from Chongqing Medical University in China were recruited in October 2024. Fifty‐three students attended a 4‐h workshop delivered in a clinical skills centre; 51 provided matched pre‐ and post‐test data. The intervention combined acute and critical care cases, mini‐lectures, video‐assisted teaching, role play, simulation, debriefing and repeat enactment of the same scenarios using TeamSTEPPS tools. Outcomes were measured using the Chinese TeamSTEPPS Teamwork Perceptions Questionnaire (T‐TPQ) and a researcher‐developed 30‐item knowledge test on teamwork and effective communication. Paired‐samples t tests were used for matched comparisons.

**Results:**

The T‐TPQ total score increased from 149.06 ± 15.18 to 159.45 ± 16.28. Significant improvements were also observed in team structure, leadership, situation monitoring, mutual support and communication. Knowledge test scores increased from 80.14 ± 18.29 to 87.33 ± 8.43.

**Conclusion:**

A brief, highly interactive TeamSTEPPS‐based workshop may improve undergraduate nursing students' teamwork perceptions and communication‐related knowledge in the short term.

**Impact:**

- What problem did the study address?
  ○ Undergraduate nursing curricula need feasible patient safety teaching approaches that explicitly target teamwork and communication before students enter complex clinical environments.
- What were the main findings?
  ○ A 4‐h workshop integrating authentic acute‐care scenarios, simulation, debriefing, and TeamSTEPPS tools improved self‐reported teamwork perceptions across all domains and improved knowledge scores.
- Where and on whom will the research have an impact?
  ○ Nursing educators may use brief, scenario‐based TeamSTEPPS workshops as scalable pre‐clinical or simulation‐centre teaching strategies, while future studies should test longer‐term behavioural transfer using objective teamwork measures.

**Reporting Method:**

This study adhered to the TREND reporting guideline.

**Patient or Public Contribution:**

No patient or public contribution.

## 1 Introduction

Patient safety remains a major challenge for healthcare systems. More than two decades after To Err Is Human, avoidable harm continues to be a central concern, and safer care depends heavily on how well healthcare teams communicate, coordinate, and respond to risk (Institute of Medicine 2000; Rosen et al. 2018). Communication failures and hesitancy to speak up remain recurrent contributors to unsafe care, particularly in complex, fast‐moving clinical environments (Okuyama et al. 2014).

Preparing students for this reality should begin before registration. The Quality and Safety Education for Nurses initiative identified teamwork and collaboration as core competencies for nursing education (Cronenwett et al. 2007). Consistent with this, studies using patient safety competence measures show that nursing students are generally more confident in technical or clinical aspects of safety than in sociocultural domains such as teamwork, risk management and speaking up, with lower confidence often reported in clinical settings (Alquwez et al. 2019; Lukewich et al. 2015). This gap is particularly concerning in acute deterioration and critical care situations, where rapid changes in patient status require clear role allocation, timely escalation and closed‐loop communication (Härgestam et al. 2013).

## 2 Background

Patient safety education can improve nursing students' knowledge, attitudes and competency‐related outcomes, although the reported effects and intervention formats remain heterogeneous. Reviews of undergraduate nursing education have highlighted active learning, supervision, feedback and simulation as useful components, but they have not established one clearly superior educational model (Bianchi et al. 2016; De Rezende et al. 2025; Kim et al. 2025).

Team Strategies and Tools to Enhance Performance and Patient Safety (TeamSTEPPS) is an evidence‐based teamwork system developed jointly by the US. Department of Defence and the Agency for Healthcare Research and Quality (AHRQ) to improve communication, coordination and patient safety in healthcare. It was developed from a substantial body of research on teamwork, team training and culture change and translates teamwork science into practical, teachable behaviours (King et al. 2008). In TeamSTEPPS 3.0, a clearly defined team structure supports four teachable and learnable skills: communication, team leadership, situation monitoring and mutual support. These skills are operationalised through structured tools such as SBAR, call‐out, check‐back, brief, huddle and debrief, and the modular curriculum can be adapted for preprofessional learners and shorter teaching formats (Agency for Healthcare Research and Quality 2023a, 2024a).

Studies involving nursing students suggest that TeamSTEPPS‐based education can improve teamwork‐related outcomes, but the interventions have generally been more extensive than a single short workshop. Greene and Doss (2021) combined TeamSTEPPS training with simulation, Karlsen et al. (2022) evaluated a longitudinal team‐training programme, Ross et al. (2021) integrated TeamSTEPPS across a baccalaureate nursing curriculum, and Maguire et al. (2015) integrated 10 h of simulation‐based TeamSTEPPS training at designated points throughout an undergraduate nursing curriculum. In China, undergraduate nursing research has more often focused on individual communication strategies; for example, an ISBARR intervention improved knowledge of and attitudes towards interprofessional communication (Wang et al. 2022).

Taken together, this literature leaves three related questions insufficiently addressed. First, evidence remains limited on whether a stand‐alone four‐hour TeamSTEPPS workshop, rather than a longitudinal or curriculum‐wide programme, can produce measurable immediate gains. Second, there is limited evidence among Chinese undergraduate nursing students for a comprehensive TeamSTEPPS intervention that addresses multiple teamwork domains rather than a single communication tool. Third, the effects of a brief intervention have not been consistently examined using both a validated measure of teamwork perceptions and a content‐specific assessment of teamwork and effective communication knowledge. The present study addresses these gaps by evaluating a 4‐h, nursing‐focused, scenario‐based TeamSTEPPS patient safety workshop and assessing changes in both teamwork perceptions and communication‐related knowledge. Accordingly, this study aimed to examine changes in undergraduate nursing students' teamwork perceptions and communication‐related knowledge following a 4‐h TeamSTEPPS‐based patient safety workshop.

## 3 The Study

The aim of this study was to examine changes in undergraduate nursing students' teamwork perceptions and communication‐related knowledge following a 4‐h TeamSTEPPS‐based patient safety workshop.

## 4 Methods

### 4.1 Design

A single‐group pretest‐posttest quasi‐experimental study was conducted.

### 4.2 Participants and Setting

Participants were third‐year undergraduate nursing students from Chongqing Medical University, Chongqing, China. Eligible students had completed foundational nursing courses, including Fundamentals of Nursing, Health Assessment, Nursing Management, Nursing Education, and related laboratory courses, and had also completed a 1‐month clinical observation placement in the university‐affiliated hospital during the previous summer. Students who had not passed earlier coursework or clinical observation requirements were excluded. A total of 53 students were recruited and attended the workshop.

### 4.3 Workshop Intervention

The workshop was delivered on 26 October 2024 in the clinical teaching and simulation centre of the First Affiliated Hospital of Chongqing Medical University and lasted 4 h. The teaching team comprised one associate professor of nursing with extensive clinical experience and two postgraduate teaching assistants. All facilitators had completed formal TeamSTEPPS training. Students were randomly allocated to five small groups before the workshop began. The 4‐h duration referred only to the educational activities; baseline and post‐intervention assessments were conducted separately and were not included in the workshop.

The workshop started with a collaborative building‐block icebreaker to surface teamwork habits and establish group engagement. It then moved into four TeamSTEPPS domains—communication, leadership, mutual support and situation monitoring—using acute and critical care crisis cases. In each module, students first enacted a brief presimulation response to a clinical case, after which the facilitators delivered focused teaching through short lectures, video‐assisted examples and discussion of positive and negative exemplars. The relevant TeamSTEPPS tools were then practised through a second, more deliberate simulation of the same case, followed by self‐reflection and facilitator feedback. Group points were used to encourage active participation throughout the session.

**FIGURE 1:**
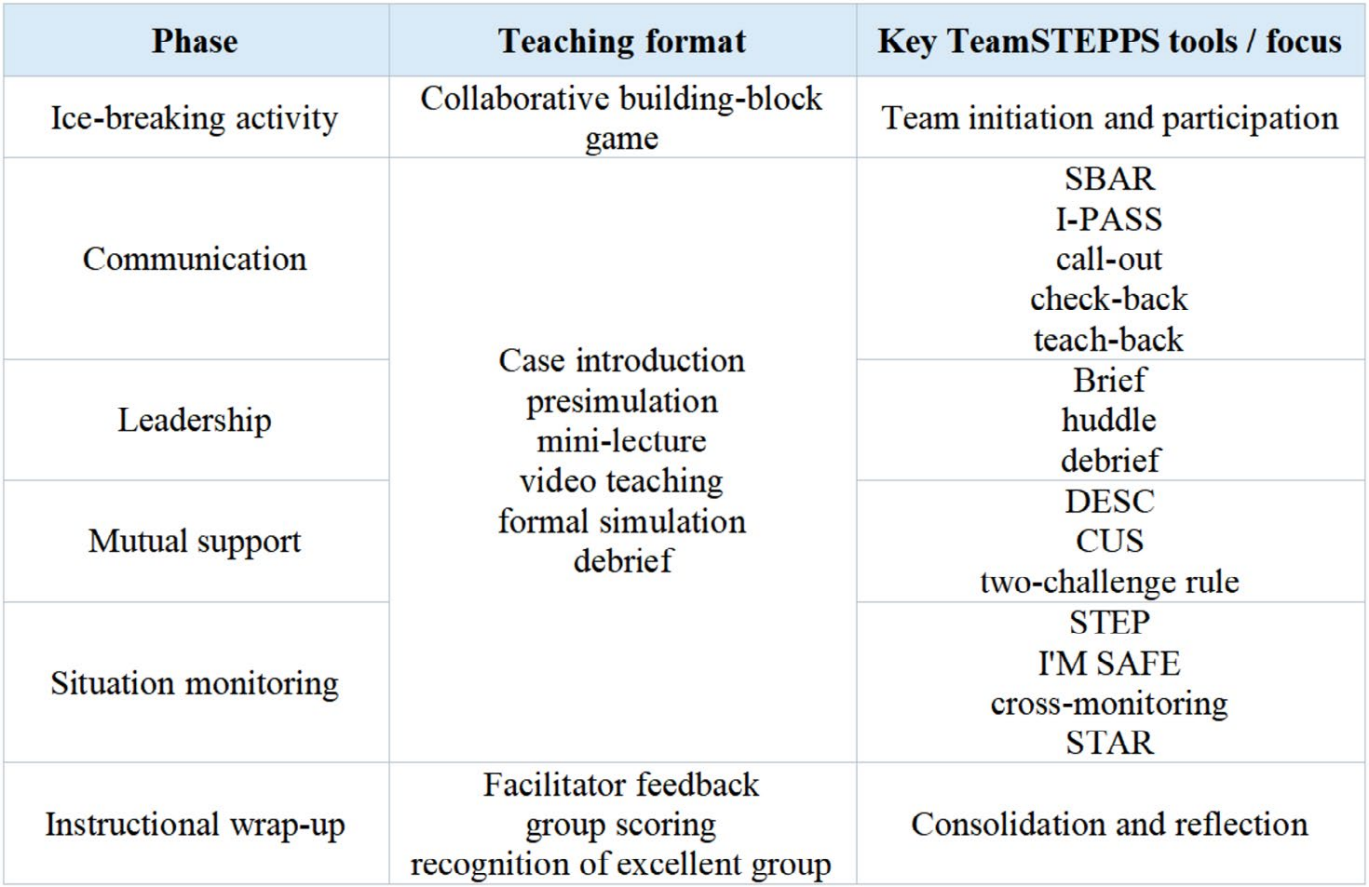
Structure and content of the TeamSTEPPS‐based patient safety workshop.

This design deliberately linked explanation, enactment, feedback and re‐enactment. Rather than only introducing terminology, the workshop asked students to apply structured communication tools (e.g., SBAR, I‐PASS, teach‐back, call‐out and check‐back), leadership processes (brief, huddle and debrief), mutual support strategies (DESC, CUS and the two‐challenge rule) and situation‐monitoring tools (STEP, I'M SAFE, cross‐monitoring and STAR) to time‐pressured scenarios (Figure 1).

### 4.4 Measures

Teamwork perceptions were assessed using the Chinese version of the TeamSTEPPS Teamwork Perceptions Questionnaire (T‐TPQ). The T‐TPQ contains 35 items across five domains—team structure, leadership, situation monitoring, mutual support, and communication—rated on a 5‐point Likert scale. Higher scores indicate more positive teamwork perceptions. The original validation study supported the measure's relationship with patient safety constructs, and the Chinese version has shown acceptable psychometric properties for use in Chinese health professional education and training (Battles and King 2010; Keebler et al. 2014; Qu et al. 2020).

Knowledge related to teamwork and effective communication was assessed using a researcher‐developed 30‐item paper‐based test comprising 25 single‐best‐answer items and 5 multiple‐response items, with a total score of 100. The content was developed by a multidisciplinary patient safety teaching team on the basis of TeamSTEPPS concepts and the case content used in the workshop. Because this test was created for local educational evaluation, it should be interpreted as a proximal learning measure rather than an established competence instrument.

### 4.5 Data Collection

Participants completed the T‐TPQ and knowledge test within the 2 weeks before the workshop and again within the 2 weeks after the workshop. These baseline and outcome assessments were research measurement procedures and were not components of the 4‐h workshop. Anonymous matching codes were used to pair pre‐ and post‐test data.

### 4.6 Data Analysis

Data processing was conducted using SPSS software. Paired *t*‐tests or the nonparametric equivalent, the Wilcoxon signed‐rank test, were used to compare outcomes before and after the intervention. When the data were normally distributed, paired *t*‐tests were applied; for non‐normally distributed data or small sample sizes, the Wilcoxon signed‐rank test was used. *A p* value < 0.05 was considered statistically significant. A standardised pre‐ and post‐test administration procedure and anonymous matching codes were used to support paired statistical analysis, and data entry accuracy was verified. Only matchable pre‐ and post‐test data were included in the paired comparisons to avoid mixing independent observations into the paired analysis.

### 4.7 Ethical Considerations

Ethics approval was obtained by the Ethics Review Committee of the First Affiliated Hospital of Chongqing Medical University (approval number: 2024–329‐01). All participants provided informed consent.

## 5 Results

### 5.1 Participant Characteristics

Of the 53 students recruited, 51 provided matched pre‐ and post‐test data and were included in paired analyses (matched response rate 96.2%). The recruited cohort was predominantly female (52/53, 98.1%), with a mean age of 20.15 years (SD 0.63; range 19–22 years). All participants were third‐year undergraduate nursing students.

### 5.2 Changes in Teamwork Perceptions and Knowledge

Teamwork perceptions improved significantly after the workshop. The T‐TPQ total score increased from 149.06 ± 15.18 at pre‐test to 159.45 ± 16.28 at post‐test, corresponding to a mean difference of 10.39 points (95% CI 5.97–14.81; *p* < 0.001; Cohen's d_z = 0.66). Significant increases were also observed in all five T‐TPQ domains. The largest standardised gains were seen in mutual support (Cohen's d_z = 0.65), communication (Cohen's d_z = 0.64) and situation monitoring (Cohen's d_z = 0.59), whereas team structure showed a smaller but still statistically significant improvement (Cohen's d_z = 0.33), as shown in Table 1.

**TABLE 1:** Comparison of teamwork perception scores before and after the workshop.

Dimension	Pre‐test (Mean ± SD)	Post‐test (Mean ± SD)	*t*‐value	Mean difference (95% CI)	Cohen's d_z	*p*
Overall score	149.06 ± 15.18	159.45 ± 16.28	4.72	10.39 (5.97–14.81)	0.66	< 0.001
Team structure	30.69 ± 3.01	32.14 ± 4.01	2.39	1.45 (0.23–2.67)	0.33	0.021
Leadership	29.59 ± 3.56	31.78 ± 3.62	3.78	2.20 (1.03 to 3.36)	0.53	< 0.001
Situation monitoring	29.75 ± 3.54	32.00 ± 3.49	4.19	2.26 (1.18–3.34)	0.59	< 0.001
Mutual support	29.29 ± 3.39	31.69 ± 3.51	4.64	2.40 (1.36–3.43)	0.65	< 0.001
Communication	29.75 ± 3.44	31.84 ± 3.22	4.57	2.10 (1.18–3.02)	0.64	< 0.001

*Note:* Mean differences, 95% confidence intervals and t values are oriented as post‐test minus pre‐test; positive values indicate higher post‐test scores. Cohen's d_z is the paired‐samples standardised mean difference. Minor discrepancies between differences calculated from the displayed pre‐ and post‐test means and the reported mean differences are due to rounding.

Knowledge scores also increased significantly after the workshop. The mean test score rose from 80.14 ± 18.29 to 87.33 ± 8.43, yielding a mean difference of 7.20 points (95% CI 1.96–12.43; *p* = 0.008; Cohen's d_z = 0.39), as shown in Table 2. Taken together, these findings indicate improvement in both self‐reported teamwork perceptions and communication‐related learning outcomes over the short term.

**TABLE 2:** Comparison of knowledge test scores before and after the workshop.

	Pre‐test (Mean ± SD)	Post‐test Mean ± SD	*t*‐value	Cohen's d_z	Mean difference (95% CI)	*p*
Knowledge test score	80.14 ± 18.29	87.33 ± 8.43	2.76	0.39	7.20 (1.96–12.43)	0.008

*Note:* Mean differences, 95% confidence intervals and t values are oriented as post‐test minus pre‐test; positive values indicate higher post‐test scores. Cohen's d_z is the paired‐samples standardised mean difference. Minor discrepancies between differences calculated from the displayed pre‐ and post‐test means and the reported mean differences are due to rounding.

## 6 Discussion

### 6.1 Principal Findings and Central Contribution

This study found that a 4‐h TeamSTEPPS‐based patient safety workshop was associated with improvements in undergraduate nursing students' teamwork perceptions across all five T‐TPQ domains and in communication‐related knowledge. The total T‐TPQ change was of moderate magnitude, with the largest standardised gains in mutual support, communication and situation monitoring, whereas the knowledge gain was smaller and team structure showed the least change. The central contribution of the study is therefore not simply that TeamSTEPPS education was beneficial, but that a brief, nursing‐focused and scenario‐based format produced measurable short‐term changes in two complementary educational outcomes: a validated measure of teamwork perceptions and a content‐specific assessment of teamwork and communication knowledge. This pattern accords with recent evidence syntheses showing that patient safety education can improve nursing students' knowledge, attitudes and competence‐related outcomes, although effects vary across interventions and outcome measures (De Rezende et al. 2025; Park and Yeom 2025).

These results should be interpreted as proximal educational effects. The single‐group design does not establish that the workshop alone caused the observed changes, and the outcomes do not demonstrate sustained clinical behaviour or improvements in patient outcomes. In Kirkpatrick terms, the study provides evidence of short‐term learning‐related change rather than behavioural transfer or clinical effectiveness (Alliger and Janak 1989). This distinction defines both the contribution and the limits of the present findings.

### 6.2 Interpretation of the Findings

The workshop design offers a plausible explanation for the observed changes. Students first responded to an acute or critical care scenario using their existing approach, then received focused instruction and video examples, practised specific TeamSTEPPS tools, participated in structured debriefing and repeated the same scenario. This sequence enabled learners to compare an intuitive response with a more structured team response and to use feedback immediately. It is consistent with experiential learning and deliberate practice, in which performance develops through action, reflection, focused feedback and renewed action (Ericsson et al. 1993; Kolb 1984). The TeamSTEPPS guidance for preprofessional students similarly emphasises short, modular teaching and active strategies such as exercises, discussion and simulation rather than passive instruction alone (Agency for Healthcare Research and Quality 2023b). These mechanisms are plausible, although the present study was not designed to test mediation. The use of structured debriefing is also supported by evidence showing that debriefing approaches can shape learning outcomes in nursing simulation (Niu et al. 2021).

The domain‐specific pattern further clarifies what a brief workshop may and may not change. Communication, mutual support and situation monitoring were repeatedly made visible through call‐outs, check‐backs, CUS, cross‐monitoring and STEP during time‐pressured scenarios. These are explicit behaviours that can be demonstrated, rehearsed and corrected within one session. By contrast, team structure includes broader understandings of roles, responsibilities, hierarchy and accountability, which are also shaped by the wider curriculum and clinical culture. Its smaller change may therefore reflect both the limited duration of the intervention and the comparatively high baseline score. Similarly, the smaller knowledge effect should be interpreted in light of high baseline performance and the use of a locally developed test. The findings suggest that a brief workshop may be particularly useful for introducing explicit and rehearsable teamwork behaviours, but less able to alter system‐level role perceptions or establish stable competence after a single exposure. This interpretation is consistent with evidence that closed‐loop communication and shared team situation awareness support coordination in time‐pressured clinical teams (Härgestam et al. 2013; Weller et al. 2024).

For patient safety education, the important implication is that teamwork concepts became linked to concrete actions for escalation, information verification, workload redistribution and speaking up. The workshop provided a shared vocabulary that students could use to recognise and discuss safety‐critical teamwork. Nevertheless, because no objective performance or clinical outcomes were assessed, the present contribution remains one of educational preparedness rather than demonstrated improvement in patient safety outcomes. This educational emphasis is clinically relevant because communication quality and mode are associated with patient safety incidents across healthcare settings (Keshtkar et al. 2025).

### 6.3 Implications for Nursing Education and Patient Safety Curricula

The four‐hour format has practical relevance because it can function as an entry module within an existing undergraduate curriculum without requiring the immediate creation of a separate semester‐long course. This interpretation is consistent with AHRQ guidance that TeamSTEPPS content for preprofessional learners can be organised in relatively short, modular segments focused on tools relevant to the learners' expected clinical situations (Agency for Healthcare Research and Quality 2023b). The workshop should, however, be viewed as an initial structured exposure rather than a complete teamwork curriculum.

Several integration points are possible. In patient safety or nursing management courses, educators could introduce team structure, speaking‐up strategies, briefs, huddles, and debriefs. In health assessment and clinical skills courses, SBAR, call‐out, and check‐back could be embedded in deterioration recognition, handover, and escalation exercises. In simulation courses, TeamSTEPPS behaviours could be written explicitly into scenario objectives, observer checklists, and debriefing prompts rather than treated as incidental communication skills. Immediately before clinical placement, a shorter refresher could rehearse handover, escalation, workload support, and challenge‐and‐advocacy behaviours using cases matched to the placement setting.

Longitudinal reinforcement is likely to be important for transfer. A practical sequence would be to introduce the common language in a brief workshop, revisit the same tools in later simulations of increasing complexity, and then prompt students to identify or use these behaviours during clinical placements. Curriculum‐wide TeamSTEPPS integration has been associated with improved nursing students' teamwork knowledge, and students exposed to longitudinal team training have described learning teamwork as a continuing process whose relevance becomes clearer through clinical experience (Karlsen et al. 2022; Ross et al. 2021). The present findings therefore support the four‐hour workshop as a feasible starting point around which repeated practice can be organised, rather than as a one‐off endpoint. Although conducted with clinical teams rather than students, a TeamSTEPPS implementation study found that targeted reinforcement was associated with improved leadership and communication behaviours after initial training (Lee et al. 2021).

Implementation also requires attention to faculty preparation and assessment. Educators would need facilitators who understand the TeamSTEPPS tools, protected small‐group practice time, clinically credible scenarios and a consistent approach to debriefing. Observable criteria could be incorporated into simulation assessment using the TeamSTEPPS Team Performance Observation Tool or a locally validated equivalent (Agency for Healthcare Research and Quality 2024b). Because this study did not measure implementation fidelity, faculty workload, cost or acceptability, it would be premature to describe the workshop as cost‐effective or universally scalable. Its practical value is that it offers a clearly bounded model that can now be tested across different educational contexts.

### 6.4 Limitations and Directions for Future Research

This study has several limitations. First, the single‐group pretest‐posttest design cannot exclude testing effects, maturation, concurrent learning, or Hawthorne effects. Second, the study involved one cohort from one institution and was predominantly female, limiting generalisability. Third, post‐testing occurred within two weeks, so the durability of the changes is unknown. Fourth, teamwork perceptions were self‐reported and the knowledge test was locally developed; neither outcome demonstrates observable performance in simulation or clinical practice. Finally, the study did not assess clinical placement performance or patient‐related outcomes. Conclusions should therefore remain at the level of short‐term educational benefit.

Future research should use controlled, preferably multi‐centre designs with an active comparison condition and explicit monitoring of intervention fidelity. Follow‐up assessments at one, three, six and where feasible, twelve months would clarify knowledge retention and the persistence of teamwork perceptions. Studies should also compare a single workshop with booster sessions or longitudinal reinforcement to determine the dose and timing required to sustain learning.

Behavioural transfer should be evaluated directly. Blinded raters could assess communication, leadership, mutual support and situation monitoring during standardised simulations using the TeamSTEPPS Team Performance Observation Tool, while structured preceptor ratings or observations could examine whether students use the tools during clinical placements (Agency for Healthcare Research and Quality 2024b). Linking educational outcomes to safety‐relevant processes, such as the completeness of handovers, escalation behaviour or recognition of communication hazards, would provide a more credible bridge between classroom learning and patient safety than self‐report measures alone.

The workshop should also be adapted and tested as interprofessional education involving nursing, medical and other health‐profession students. Contemporary interprofessional competencies emphasise shared communication, role clarification, teamwork and collaborative decision‐making, and TeamSTEPPS‐based simulation has shown potential for developing these abilities in mixed student teams (Interprofessional Education Collaborative 2023; Paige et al. 2017). Comparative studies could determine whether interprofessional delivery produces greater behavioural transfer than nursing‐only delivery. Finally, implementation studies should examine acceptability, faculty workload, resource requirements, fidelity and cost to determine whether the four‐hour model can be reproduced across institutions with different simulation capacity.

## 7 Conclusion

A 4‐h TeamSTEPPS‐based patient safety workshop was associated with short‐term improvements in undergraduate nursing students' teamwork perceptions and communication‐related knowledge. Its central educational contribution is to show that a brief, highly interactive and scenario‐based format can serve as an initial point of entry for explicit teamwork and patient safety training, particularly before clinical placement or within simulation‐based courses. The workshop should be reinforced longitudinally rather than treated as a stand‐alone solution. Controlled multi‐centre studies are now needed to evaluate long‐term retention, objective teamwork behaviour in simulation and clinical placements, and interprofessional adaptations involving nursing, medical and other health‐profession students.

## Author Contributions

**Yunrui Yin:** conceptualization, methodology, data curation, validation, formal analysis, visualization, investigation, writing – original draft, writing – review and editing. **Weirong Tang:** data curation, validation, investigation, formal analysis. **Yuerong Li:** conceptualization, funding acquisition, supervision, writing – review and editing, project administration. **Huilin Ye:** data curation, validation, investigation, formal analysis. **Yingjia Zhang:** investigation, data curation, validation. **Xiaorong Wu:** data curation, investigation, validation. **Lulu Wan:** validation, data curation. **Enqi Huang:** validation, data curation.

## Funding

This research was supported by the First Affiliated Hospital of Chongqing Medical University Fund (HLZD2024‐04).

## Conflicts of Interest

The authors declare no conflicts of interest.

## Data Availability

The data that support the findings of this study are available from the corresponding author upon reasonable request. This study did not involve the collection or utilisation of genetic resources as defined by the Nagoya Protocol; therefore, no fieldwork permits were applicable. The authors affirm that the methods used in the data analyses are suitably applied to their data within their study design and context, and the statistical findings have been implemented and interpreted correctly. The authors agree to take responsibility for ensuring that the choice of statistical approach is appropriate and is conducted and interpreted correctly as a condition to submit to the Journal.
